# EVA and SEBS-MA copolymers incorporated silicone rubber/SEBS blends: improvement of mechanical and thermal properties

**DOI:** 10.1038/s41598-023-49796-6

**Published:** 2023-12-18

**Authors:** Ehsan Alikhani, Mohsen Mohammadi

**Affiliations:** Department of Polymer Engineering, Qom University of Technology, Qom, 3718146645 Iran

**Keywords:** Polymers, Mechanical properties, Polymer characterization

## Abstract

Enhancing the mechanical and thermal properties of Silicone rubber (SR)/SEBS blends using various compatibilizers opens the opportunity for such new blends to meet the market desire. For this purpose, blends with a 1:1 ratio of SR and SEBS are prepared with different amounts of EVA or SEBS-MA copolymers as compatibilizer. Mechanical properties of the blend are enhanced by adding EVA and SEBS-MA. Addition of 6 phr EVA profoundly improves the tensile strength from 7.70 to 10.06 MPa. Thermogravimetric analysis reveals that the presence of compatibilizer can improve the thermal stability of the blend, especially its initial degradation temperature (T_5%_). T_5%_ of the blend increases from 376 to 390 °C when comprising 6 phr SEBS-MA. Morphology of the blends is investigated using SEM and AFM. Results of the relaxation modulus curves obtained by rubber process analyzer (RPA) demonstrate that the amount of relaxation in the uncured blends is higher than the cured ones. A comparison of the relaxation of the blends indicates that the relaxation modulus of the SEBS-MA compatibilized blends is enhanced more than other blends after curing. Further investigations indicate that the compatibilized blends exhibit higher tear energy and lower compression set.

## Introduction

Nowadays, the amount of use of polymers has surpassed other materials due to their light weight, corrosion resistance, accessibility, and easy processability^1,2^. Nevertheless, polymers also have limitations, such as low mechanical strength. Such limitations can be overcome by using appropriate methods^3–9^. Polymer blending is considered one of the most important methods to improve the properties of polymers. Synthesis of a new polymer involves significant costs; in contrast, via combination of two or more structurally different polymers, simply and at low cost, a novel material can be obtained^10,11^. The performance of polymer blends is highly dependent on the interfacial interactions and compatibility between phases. Most of polymer blends are immiscible, and have no proper interaction between their phases. Adding copolymers or making them during (reactive) blending are among the main methods of improving the compatibility of polymer blends^12,13^.

Essabir et al.^14^ used SEBS-MA copolymer to raise the compatibility of ABS/PA6 blend. The presence of SEBS-MA improved the adhesion between phases and thus incremented the tensile strength of the compatibilized blends. Hellati et al.^15^ used ethylene–vinyl acetate copolymer (EVA) as a compatibilizer to augment the compatibility of PET/HDPE blends. Khanra et al.^16^ used SR-MA graft copolymer to boost the compatibility between FKM blend and SR. They found that the tensile strength of the compatibilized blends raised by 71% compared to the un-compatibilized blends. In another work, Khanra et al.^17^ showed that the tensile strength of FKM/SR blends improved by 21% in the presence of FKM-g-acrylamide graft copolymer.

SR or polydimethylsiloxane (PDMS) has unique properties such as chemical inertness, high heat resistance, abrasion resistance, excellent ozone and oxidation resistance, low toxicity, weatherability resistance, biocompatibility, and extremely low-temperature flexibility due to its special molecular structure that originates from Si–O bonds^18–21^. Nonetheless, the poor mechanical properties of SR have limited its applications. To overcome this drawback, SR is usually blended or copolymerized with other polymers^22–24^. Poly (styrene-ethylene butylene-styrene) triblock copolymer (SEBS) is one of the most important conventional thermoplastic elastomers (TPEs), which has good thermal stability, aging resistance, and electrical properties. Furthermore, weatherability resistance, good resistance to humidity and hydrolysis, and high elasticity are among the other properties of this copolymer that has made it find wide industrial applications^25–28^.

To the best of our knowledge, the effect of compatibilizer on the properties of SR/SEBS blend has not been investigated. In our previous work^29^, for the first time, a novel polymer blend of SR/SEBS with various compositions was prepared, which indicated good mechanical, thermal and, rheological features. For instance, the presence of 25% SEBS could increase the tensile strength of SR from 5.66 to 7.56 MPa. Potential areas of application of SR/SEBS blends could be the across wire and cable industries, and soft-touch appliances. These blends can be used in the matrix of dielectric elastomers in actuators, sensors, biomedicine, and energy collectors. Moreover, SR/SEBS blends could be utilized in aerospace, transportation, and petroleum equipment manufacturing.

The purpose of this study is to investigate the effect of EVA and SEBS-MA copolymers on the behavior and compatibility of SR/SEBS blends. The morphology, cure and rheology behavior, mechanical and thermal properties of the compatibilized blends are characterized. This is the first time that the effect of the type and amount of compatibilizers on the properties of SR/SEBS blends has been studied. A comprehensive investigation of the properties of such blends through incorporating compatibilizer would be a worthy assessment to spread the application of such intriguing blends in various fields.

## Experimental

### Materials

Commercial SR (NE-5280) with a density of 1.25 g/cm^3^ obtains from Djsilicone Co., Ltd. (China). Commercial Tri-block copolymer SEBS (Globalprene-7550U), with 30 wt% styrene units and a density of 0.91 g/cm^3^, is supplied by LCY Co., Ltd (Taiwan). EVA (ES28005) including 28% vinyl acetate and a density of 0.95 g/cm^3^ is supplied LG chem. SEBS-MA (FG1901) with 30 wt% styrene units and 1.8% maleic anhydrate is supplied by Kraton Co., Ltd. Peroxide curing agent BIPB (Bis (tert-butylproxy isopropyl) benzene) with a purity (percent assay) of 97% is from Rhein Chemie Co., Ltd (China).

### Preparation of the samples

**Figure 1:**
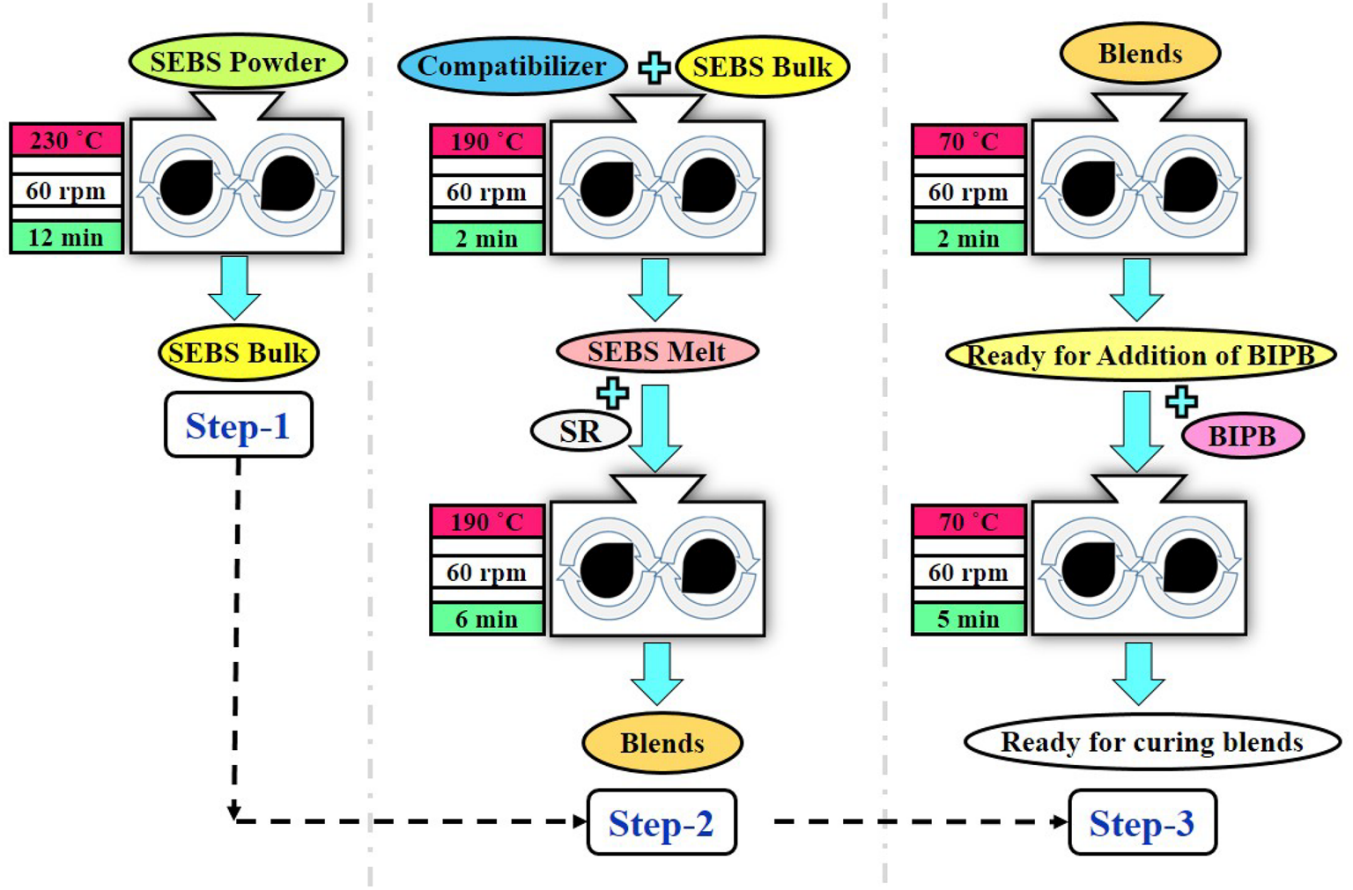
Scheme of blends preparation by using internal mixer.

**Figure 2:**
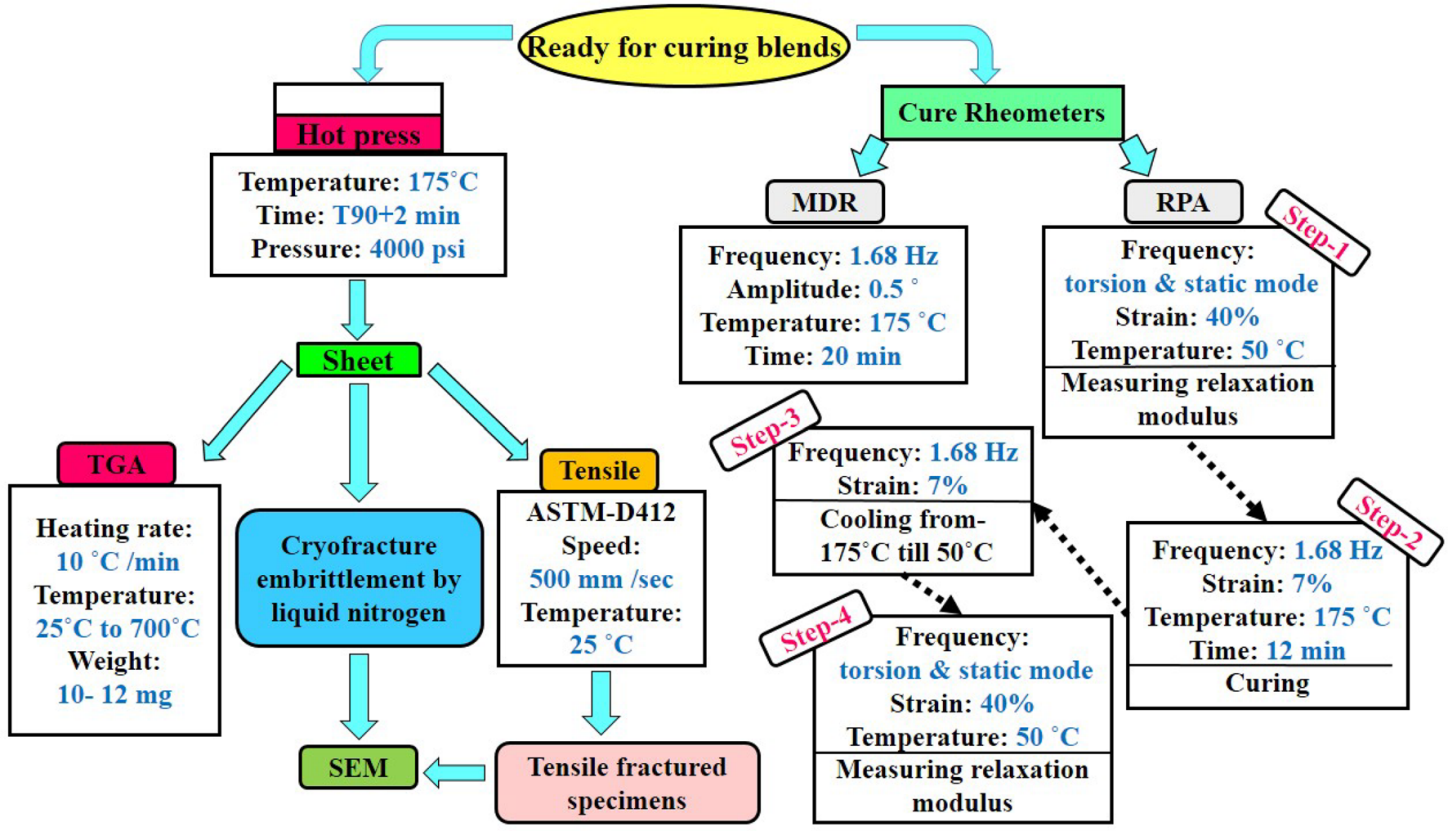
Simple flow diagram of testing procedures and conditions.

As-received SEBS for this study is in powder form. Processing of the powder polymer is difficult due to the difference between the bulk density and the pour density, as well as poor heat transfer by the powder particles. In order to facilitate the processing and to improve the mixing as well as to lower the blending temperature, all SEBS is first processed at once. The SEBS powder is fed into a Rheosens Lab Torque Rheometer, IPPI Co., Ltd (Iran) as an internal mixer, with a mixing chamber volume of 60 cm^3^ and maintaining a fill factor of 0.75 to give enough space for proper mixing. The mixing process is carried out at 230 °C at a rotor speed of 60 rpm for 12 min (Step-1). SEBS is melted in the last 1.5 min, but before that, it is still in powder form with virtually no stress on its polymer chains. SR and SEBS are blended in the internal mixer as per the recipe demonstrated in Fig. 1. The blending is done at 190 °C at a rotor speed of 60 rpm for 8 min. Formulations of all the blends are given in Table 1. Formerly, SEBS and compatibilizer are fed into the chamber of the internal mixer, and it melts after 2 min. Subsequently, SR is added and mixed for 6 min to prepare the blends (Step-2). The blends are mixed with peroxide curing agent (BIPB) for 5 min at 70 °C and 60 rpm (Step-3). The curing agent at the chosen temperature of mixing (70 °C) does not decompose. In the end, the ready blends for curing are used to perform various analyses in different methods (depicted in Fig. 2).

**Table 1:** Formulation of the blends.

Sample name	SEBS (wt %)	SR (wt %)	SEBS-MA (phr)	EVA (phr)
B	50	50	0	0
B/3S-MA	50	50	3	0
B/6S-MA	50	50	6	0
B/3E	50	50	0	3
B/6E	50	50	0	6
Pure SEBS	100	0	0	0
Pure SR	0	100	0	0
SEBS/6S-MA	100	0	6	0
SEBS/6E	100	0	0	6
SR/6S-MA	0	100	6	0
SR/6E	0	100	0	6

### Cure characteristics

The cure characteristics of the blends are studied by a moving die rheometer (MDR) (SMD-200, Santam Co., Ltd., Iran). About 5 cm^3^ of samples are used to perform the experiment. The test is executed at a frequency of 1.68 Hz, a temperature of 175 °C, and an amplitude of 0.5° for 20 min. Three repetitions of the test are performed for each blend and average values are reported. The cure rate of the blends is calculated as follows^30^:

1 $$ {\text{Cure rate}} = \frac{{{\text{Optimum cure time torque}} - {\text{t}}_{{{\text{s2}}}} {\text{ torque}}}}{{{\text{Optimum cure time}} - {\text{t}}_{{{\text{s2}}}} \, }} $$

### Measurement of mechanical tensile properties

The mechanical tensile properties are evaluated by an electronic tensile machine (STM-20, Santam Co., Ltd., Iran) with a tensile speed of 500 mm/min, according to standard ASTM-D412. For each blend, five similar specimens are tested, and the average values of tensile strength, strain at break, modulus value at 50% strain (M50), and modulus value at 100% strain (M100) are evaluated. Also, the crosslink density (CLD) of the blends is calculated using the M50 and with the help of the Mooney–Rivlin equation^31^ as follows:

2 $$ \sigma = \nu RT\left( {\lambda - \frac{1}{{\lambda^{2} }}} \right) $$

In this equation, σ is the modulus, ν is the CLD, λ represents the extension ratio, R indicates the gas constant, which is 8.314 (J/ mol K), and T is the absolute temperature during the test (here, 298 K).

### Thermogravimetric analysis (TGA)

The thermal degradation behavior of the blends is determined by a Thermogravimetric analyzer (TGA-SF1, Mettler Co., Ltd., Switzerland). The heating rate is 10 °C/min. A weight of 10–12 mg of specimens is used, and the analysis is done from ambient temperature to 700 °C in a nitrogen atmosphere. For each blend, three similar specimens are tested and the average values are calculated.

### Morphology of the blends

Tensile fractured and cryo-fractured surfaces of the blends are investigated using a VEGA-II (Tescan Co., Ltd., Czech Republic) Scanning Electron Microscopy (SEM) at an acceleration voltage of 20 kV. The fractured surface of specimens is sputter-coated with a thin gold layer in a vacuum chamber for conductivity before the test. In addition, surfaces of the blends are examined with atomic force microscopy (AFM), universal SPM (Ambios Technology, USA) under tapping mode. In this test, silicon cantilever is at a resonant frequency of 180 kHz.

### Measuring the relaxation modulus of the blends

Viscoelastic properties of rubbers can be investigated by studying values such as relaxation modulus. The values are measured by the rubber process analyzer (RPA) instrument. The rubber process analyzer (RPA 2000) from Alpha Technologies Co. (UK) is used to study the relaxation modulus of the uncured and cured blends. About 5 cm^3^ of blends are used to perform the tests. The device cavity is closed under high pressure during the test to decline the wall slip. This experiment is done under four steps (Fig. 2). At first, the relaxation modulus of uncured blends is measured at a temperature of 50 °C, and 40% strain. In the second step, the blends are cured at 175 °C, 7% strain, and 1.68 Hz frequency for 12 min, then they are cooled to 50 °C. In the fourth step, the relaxation experiment is done on cured blends with the same conditions as the first step.

### Tear resistance experiment

Tearing energy of the blends is measured according to ASTM D 624 for the type T test configuration using trouser tear test specimens at room temperature and cross-head speed of 500 mm/min via an Instron universal testing machine (model 6025). The experiment is repeated for five specimens of each of the blends and average values are reported.

### Measurement of compression set

The compression set experiment (ASTM D 395) was conducted on a standard test specimen of cured blends in cylindrical shape with dimensions of 20 mm diameter and 15 mm thickness. The test specimens were mounted between the plates of the compression device, with spacers on both sides. The compression percentage used was 25% of the original thickness of the samples. Then, the compressor device, with the assembled specimen inside, was placed in the oven at 75 °C for 72 h. Upon completion of the testing, the test specimens were removed and allowed to cool for 30 min. Then, the final thickness of the sample was measured to determine its compression set. For each blend, five similar specimens are tested and the average values are calculated.

## Results and discussion

### Cure characteristics

**Figure 3:**
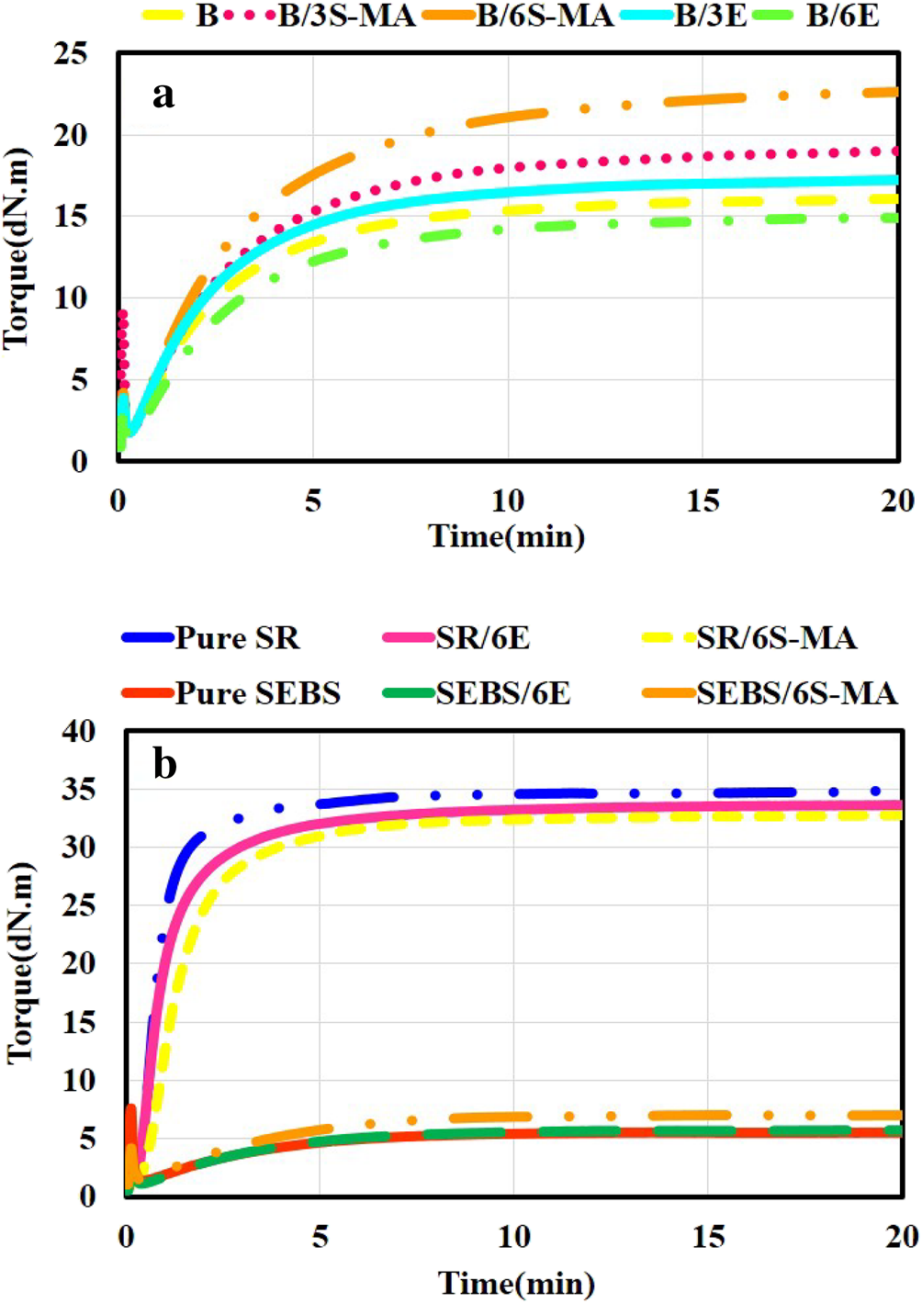
Time dependency of torque for the blends and pure polymers during curing obtained from MDR.

The rheograms of blends are shown in Fig. 3. The curves of all blends indicate an initial decline in torque. The initial reduction in torque is due to the softening of the polymers while heating. The softening of the polymers leads to a decrease in viscosity and, as a result, a reduction in the torque required to oscillate the rheometer disc. Next, with the activation of the curing agent and the formation of the first crosslinks, the resistance of the chains to the movement increases and increments the torque. In the end, the torque reaches a constant value. The information from Fig. 3, as T_s2_, T_90_, M_L_, M_H_, and ∆M is compiled in Table 2.

**Table 2:** Cure characteristics of the blends and pure polymers.

Name	T_s2_ (min)	T_90_ (min)	M_L_ (dN m)	M_H_ (dN m)	∆M (dN m)	Rate of cure (dN m/min)
B	0.78 ± 0.04	7.12 ± 0.20	1.71 ± 0.07	16.11 ± 0.8	14.40	1.72
B/3S-MA	0.75 ± 0.05	7.83 ± 0.19	1.79 ± 0.06	19.03 ± 0.6	17.24	1.91
B/6S-MA	0.72 ± 0.06	8.70 ± 0.17	1.87 ± 0.07	22.60 ± 0.8	20.73	2.08
B/3E	0.72 ± 0.05	6.95 ± 0.16	1.76 ± 0.08	17.21 ± 0.7	15.45	1.91
B/6E	0.87 ± 0.06	7.45 ± 0.15	1.51 ± 0.05	14.92 ± 0.6	13.41	1.51
Pure SEBS	2.50 ± 0.10	8.01 ± 0.18	1.35 ± 0.06	5.53 ± 0.3	4.18	0.35
Pure SR	0.38 ± 0.02	2.22 ± 0.11	1.25 ± 0.04	34.91 ± 0.9	33.66	15.43
SEBS/6S-MA	2.93 ± 0.12	6.97 ± 0.14	1.40 ± 0.05	6.99 ± 0.7	5.59	0.51
SEBS/6E	2.23 ± 0.10	7.02 ± 0.15	1.12 ± 0.04	5.67 ± 0.5	4.55	0.40
SR/6S-MA	0.50 ± 0.03	3.65 ± 0.13	1.15 ± 0.04	32.80 ± 0.8	31.65	4.28
SR/6E	0.37 ± 0.02	3.20 ± 0.12	1.70 ± 0.06	33.60 ± 0.9	31.91	4.02

According to Fig. 3a and the information in Table 2, the following results are inferred about the compatibilized blends with SEBS-MA:

- Adding SEBS-MA and raising its amount cause a slight increment in the cure time and cure rate of compatibilized blends compared to B blend.
- The presence of SEBS-MA has caused a slight change in the scorch time (t_s2_) of B/3S-MA and B/6S-MA blends compared to B blend.
- Adding SEBS-MA and increasing its amount elevate the maximum torque and ∆M of the SEBS-MA compatibilized blends compared to B blend.

Modification of hydrogenated polymer chains such as SEBS with materials such as maleic anhydride increases chain reactivity^32^. Pavlovsky and Siegmann^32^ used SEBS-MA as the primary substrate to improve the curing characteristics of SEBS. Therefore, it can be expected that the addition of SEBS-MA to the blends can have a positive effect on the cure behavior of the SEBS in the blends.

To better understand the effect of this compatibilizer on the curing of pure polymer components, Pure SEBS and Pure SR can be compared with SEBS/6S-MA and SR/6S-MA blends. Figure 3b and Table 2 indicate the rheograms and information obtained from them, respectively. Comparing the cure behavior of Pure SEBS and SEBS/6S-MA, it is clear that the presence of SEBS-MA in SEBS raises ∆M, crosslink density, and its cure rate. The improving effect of SEBS-MA on the cure behavior of Pure SEBS can be justified due to the higher reactivity of SEBS-MA compared to pure SEBS. In this way, SEBS-MA is well dispersed in the pure SEBS, and with its greater reactivity, it causes better distribution and more crosslinks in the SEBS polymer. Meanwhile, the comparison of Pure SR and SR/6S-MA shows that the addition of SEBS-MA caused a decrease in ∆M and also the cure rate of SR. The cure rate and ∆M of SR are much higher than that of SEBS^29^. Consequently, despite the difference in the cure rate and ∆M of SEBS and SEBS-MA, it is expected that the cure rate and ∆M of SR are still higher than that of SEBS-MA. Therefore, it is evident that adding SEBS-MA to SR will decrease its cure rate and ∆M. Moreover, it can be stated that the enhancement of cure rate and ∆M in B/3S-MA and B/6S-MA blends is related to the improvement effect of SEBS-MA on both the cure behavior of the SEBS component and the interphase of the blends.

Figure 3a, and Table 2 indicate the rheograms and curing characteristics of the EVA compatibilized blends compared to the B sample, respectively. According to this information, the following results are inferred about EVA compatibilized blends:

- The addition of EVA has caused changes in the T_s2_ of B/3E and B/6E blends compared to B sample. The presence of 3 phr EVA causes a small decrease, and the presence of 6 phr EVA causes a small increase in the T_s2_ and cure time of the blends.
- Adding 3 phr EVA causes a low increment in the cure rate of B/3E blend compared to B blend. While the addition of 6 phr EVA reduces the cure rate of B/6E compared to B and B/3E.
- The presence of 3 phr EVA slightly increases the maximum torque and ∆M of B/3E blend compared to B blend. However, the presence of 6 phr EVA decreases the maximum torque and ∆M of B/6E blend compared to B and B/3E.
- The effect of adding 3 phr EVA on curing characteristics shows a similar trend to the effect of adding SEBS-MA to the blends. However, the increase of EVA content in B/6E blend indicates a behavior contrary to the trend observed for B/3S-MA, B/6S-MA, and B/3E blends.

The vinyl acetate polar groups in the EVA chains give this polymer higher chemical activity than HDPE, LDPE, and LLDPE^33^. As respects SEBS does not have polar groups, the chemical activity and reactivity of EVA are definitely higher than SEBS. Furthermore, an olefinic nature, and a structure close to LLDPE can be imagined for the soft phase of SEBS. Passaglia et al.^34^ also prepared a blend of polystyrene and LLDPE to simulate the thermal degradation of SEBS and investigate it. In that blend, polystyrene played the role of the hard phase, and LLDPE played the role of the soft phase. Considering the fact that the chain of EVA is also made of polyethylene, it is expected that there is good compatibility between EVA and the soft phase of SEBS. Therefore, it can be considered that the addition of EVA, like the addition of SEBS-MA to blends, inspires a positive effect on the cure behavior of SEBS in the blends, apart from the compatibility issue. The results also justified such effect on the cure rate and ∆M of B/3E blend. However, B/6E blend shows unexpected behavior. The lower ∆M of B/6E blend than B and B/3E may be because EVA is a softer copolymer than SEBS copolymer, and its higher amount can reduce the stiffness of the final blend.

To better understand the effect of EVA compatibilizer on the curing of pure components, Pure SEBS and Pure SR can be compared with SEBS/6E and SR/6E blends. Figure 3b and Table 2 show rheograms and the information obtained from them, respectively. Comparing the cure behavior of Pure SEBS and SEBS/6E, it is clear that the presence of EVA in SEBS causes a very slight increase in ∆M and its cure rate. It also reduces the cure time and T_s2_ of Pure SEBS. Meanwhile, the addition of EVA has caused a decrease in ∆M and also the cure rate of SR. It also increases the cure time by 1 min. This behavior has also been observed by Ganesh et al.^30^ for SR/EVA blends cured with benzoyl peroxide (BP) and Dicumyl peroxide (DCP).

### Tensile properties

**Figure 4:**
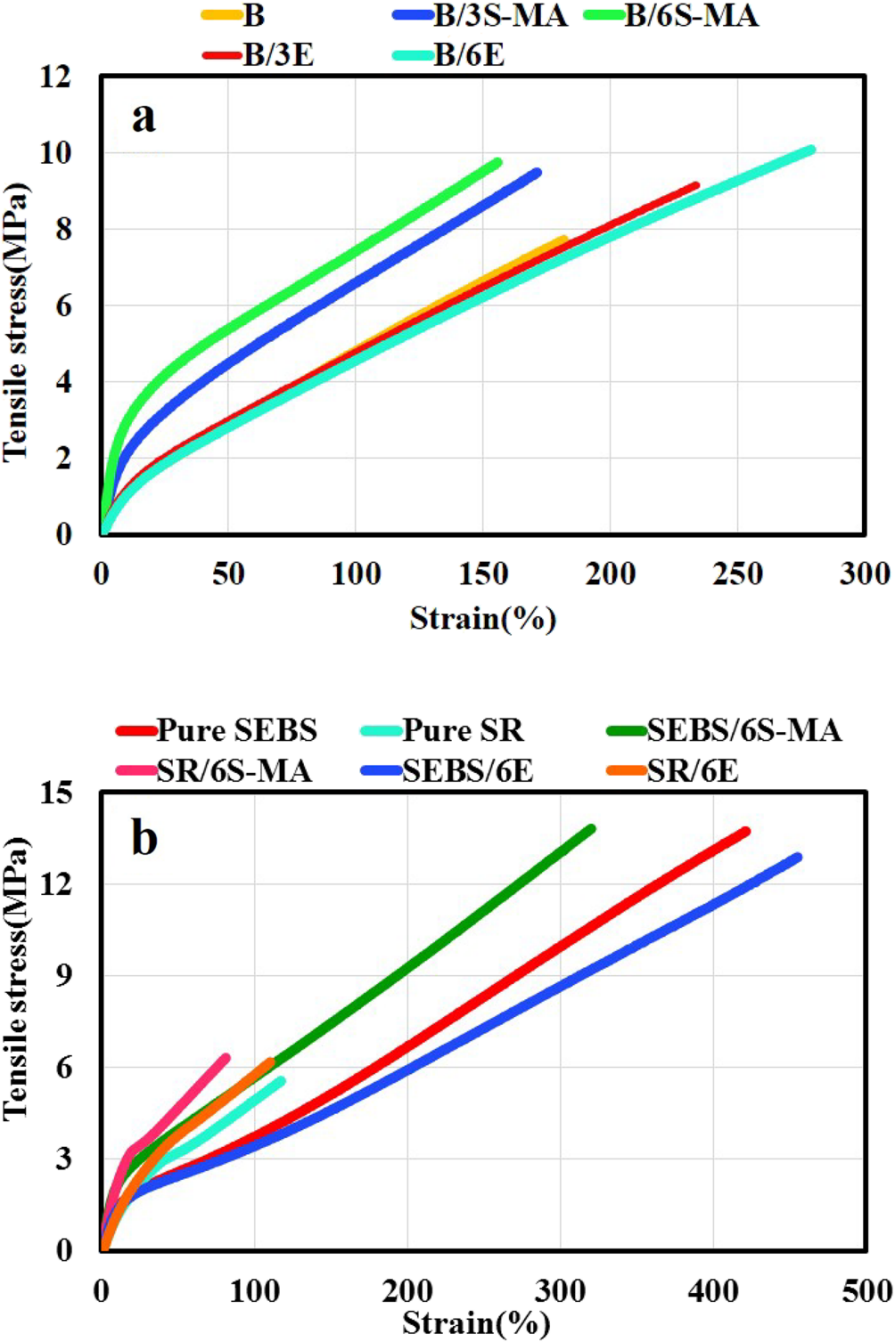
The tensile-strain curves of (**a**) B compared to compatibilized blends, (**b**) pure polymers compared to blends.

Figure 4a and Table 3, respectively exhibit the stress–strain curves and the information for the compatibilized blends compared to B blend. According to this information, the following conclusions are drawn about the SEBS-MA compatibilized blends:

- The presence of SEBS-MA significantly increased the CLD of the compatibilized blends compared to B sample.
- The addition of SEBS-MA has improved the modulus and tensile strength of B sample.
- The B/3S-MA with 3 phr compatibilizer has a lower modulus than the B/6S-MA with 6 phr compatibilizer.
- There is no significant difference between the tensile strength of B/3S-MA and B/6S-MA blends. However, the strain at break of B/3S-MA is higher than that of B/6S-MA (as obtained at 172 and 156%, respectively).

**Table 3:** The mechanical properties of the blends and pure polymers.

Name	Tensile strength (MPa)	Strain at break (%)	M50 (MPa)	M100 (MPa)	Crosslink density (ν) × 10–4 (Mol cm^−3^)
B	7.70 ± 0.6	178 ± 26	2.93 ± 0.1	4.92 ± 0.4	11.16
B/3S-MA	9.24 ± 0.8	172 ± 10	4.45 ± 0.5	6.49 ± 0.6	16.94
B/6S-MA	9.66 ± 1.5	156 ± 22	5.20 ± 0.2	7.47 ± 0.2	19.80
B/3E	9.13 ± 0.4	233 ± 30	2.83 ± 0.1	4.80 ± 0.4	10.78
B/6E	10.08 ± 0.5	277 ± 35	2.67 ± 0.3	4.77 ± 0.5	10.17
Pure SEBS	13.56 ± 0.8	423 ± 43	2.43 ± 0.4	3.70 ± 0.6	9.25
Pure SR	5.66 ± 1.3	116 ± 38	3.40 ± 0.2	5.46 ± 0.1	12.95
SEBS/6S-MA	13.75 ± 0.9	322 ± 20	3.88 ± 0.5	5.59 ± 0.4	14.77
SEBS/6E	12.95 ± 0.5	449 ± 18	2.30 ± 0.1	2.92 ± 0.2	8.76
SR/6S-MA	6.22 ± 0.6	83 ± 17	4.52 ± 0.4	-	17.21
SR/6E	6.17 ± 0.8	115 ± 28	3.63 ± 0.6	5.53 ± 0.5	13.82

Previously, it was seen that adding methacrylic acid (MAA)-grafted EPDM copolymer as a compatibilizer to the EPDM and SR blend reinforced the mechanical properties of the blend^35^. In the present work, SEBS-MA has played such a role. According to the results obtained from the MDR test, it was seen that the addition of SEBS-MA to the blends improves and optimizes the curing process of the SEBS phase of blends. As mentioned in Table 2, the ∆M of blends increments as the SEBS-MA content increases. The trend in the obtained ∆M values for the blends is confirmed by CLD calculated from the tensile test, as indicated in Table 3.

Greater modulus of B/3S-MA and B/6S-MA blends than B, as well as greater modulus of B/6S-MA than B/3S-MA, can be straightforwardly attributed to CLD degrees of these blends. Moreover, the tensile strength of the blends has also improved in the presence of SEBS-MA. The rising CLD of SEBS as well as the enhancement of compatibility between SEBS and SR in the presence of SEBS-MA, can be described as the reasons for this observation. The presence of SEBS-MA also reduces the strain at break of the blends slightly. This decrease can be associated with increased CLD and consequently incremented restriction of chain movement.

Figure 4 compares the mechanical properties of Pure SEBS and Pure SR with SEBS/6S-MA and SR/6S-MA, respectively. As can be seen, the addition of SEBS-MA has improved the modulus and tensile strength of the SEBS. However, it has declined its strain at break. This behavior is due to the growth of SEBS crosslink density in the presence of SEBS-MA. This result is in accordance with the data obtained on the cure characteristics from MDR data. Likewise, adding SEBS-MA to SR has the same effect on the mechanical properties as mentioned above. In addition, the presence of SEBS-MA, along with rising tensile strength and modulus of SR, has decreased its strain at break. A similar effect on the mechanical properties was reported in our previous work^29^ by adding 10% SEBS to SR. The blend SR/6S-MA has 6 phr SEBS-MA, equivalent to 5.66% SEBS-MA. Given the more effective curing of SEBS-MA than SEBS, the influence of 5.66% SEBS-MA on the mechanical properties of SR can be compliant with the influence of adding 10% SEBS to SR.

According to Fig. 4a, and Table 3, the following results are inferred about the EVA compatibilized blends:

- Adding EVA to SR/SEBS blend increases tensile strength and strain at break.
- The presence of EVA and increasing its content reduces the CLD and their modulus slightly.
- B/6E has better mechanical properties than blend B/3E.
- EVA is a better compatibilizer than SEBS-MA.

It could be said that the SR/SEBS blends are almost analogous to SR/EPDM blends, because soft ethylene butylene blocks in SEBS such as EPDM have an olefinic nature. Koll et al.^36^ reported that adding EVA has a positive effect on compatibilization of SR/EPDM blends and improves the mechanical properties of the blends, which is comparable to the EVA effect in the present work.

In Fig. 4b, and Table 3, the mechanical properties of SEBS/6E and SR/6E blends are compared with the mechanical properties of Pure SEBS and Pure SR blends, respectively. A comparison of SEBS/6E and Pure SEBS indicates that adding 6 phr EVA to SEBS slightly reduces SEBS modulus and tensile strength. However, it has somewhat improved the strain at break. These observations can be attributed to a slight reduction of CLD in the blends. Besides, the softness of the EVA can also play a role in decreasing the modulus and increasing the SEBS/6E strain at break compared to Pure SEBS. The comparison of SR/6E and Pure SR demonstrates that adding 6 phr EVA to SR raises its tensile strength lightly. Furthermore, the presence of the EVA has incremented the SR modulus to a small amount, and its strain at break has almost unchanged.

Changes in the SR mechanical properties in the presence of EVA are related to the enhancement effect of EVA. This improvement effect has been reported by Ganesh et al.^30^. The small efficiency of EVA is related to its low content in the SR/6E blend. Studying the effect of EVA on SR and SEBS and comparison with its effect on B blend revealed that the EVA has been well able to play a compatibilizer role in improving the compatibility of both phases in the blend.

Table 3 exhibits that in SEBS/6E sample, EVA has reduced the CLD of SEBS. It is also seen that in SR/6E sample, EVA has raised the CLD of SR. Moreover, in EVA compatibilized blends, the presence of EVA and increasing its content have decreased the CLD of the blends. Therefore, comparing the CLD of the blends, it can be expressed that for EVA compatibilized blends, the reducing effect of EVA on the CLD of SEBS component overcomes its rising influence on the CLD of the SR component.

To conclude, the influence of SEBS-MA on the mechanical properties of blends was primarily due to its positive effect on cure behavior and raising their CLD. However, the impression of EVA on the mechanical properties of the blends has been primarily due to its positive effect on improving compatibility. Because, despite the lower CLD of B/3E and B/6E blends than B blend, EVA compatibilized blends have provided better mechanical properties than B blends.

### Thermogravimetric analysis

**Figure 5:**
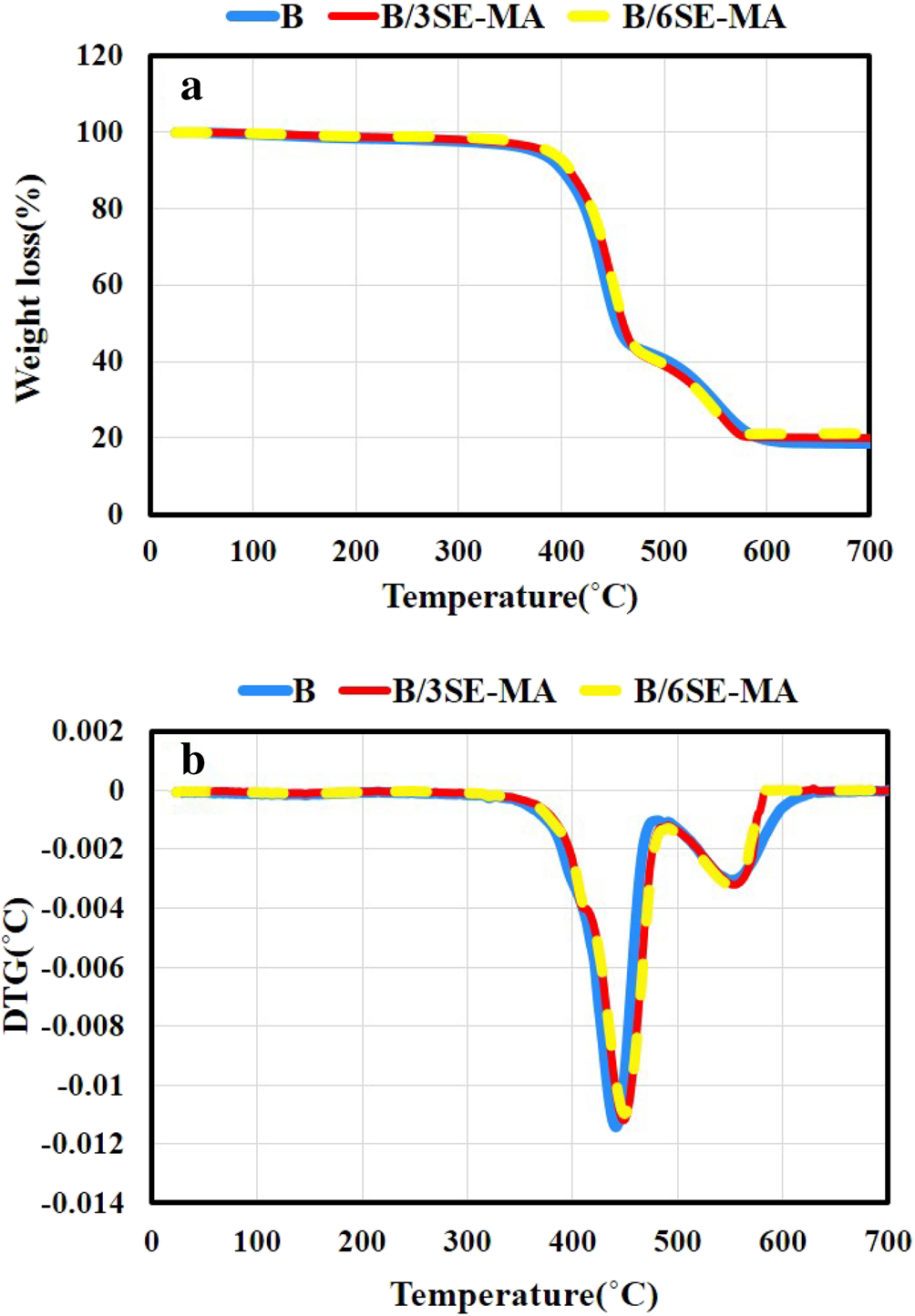
The thermograms of SEBS-MA compatibilized blends compared to B: (**a**) TGA, (**b**) DTG.

**Figure 6:**
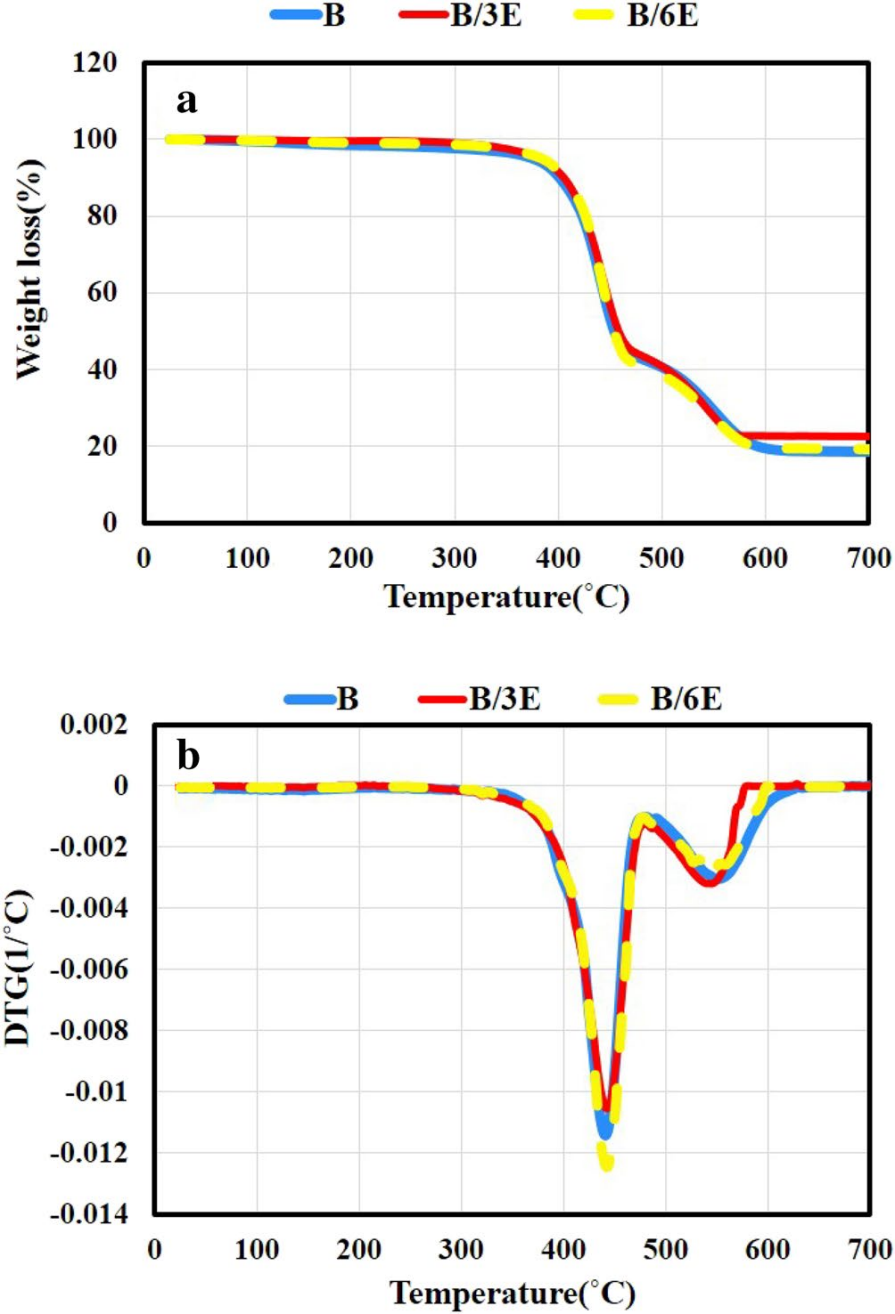
The thermograms of EVA compatibilized blends compared to B blend: (**a**) TGA, (**b**) DTG.

Figures 5a and 6a illustrate the TGA curves of the compatibilized blends compared to B blend from ambient temperature to 700 °C. Figures 5b and 6b also show the derivative thermogravimetry (DTG) curves. Moreover, data obtained from these curves have been reported in Table 4 for better comparison. The results obtained from Table 4 indicate that the presence of SEBS-MA and EVA as compatibilizers, has been able to improve all the thermal stability parameters of blends. Also, it is understood that SEBS-MA has more improvement effects than EVA. For example, the presence of 6phr SEBS-MA in B/6S-MA causes increasing T_5%_ by 14 °C compared to B blend. This is while the presence of the same amount of EVA has improved T_5%_ of B/6E blend by 9 °C compared to B blend. Much recent evidence has highlighted that adding a suitable compatibilizer can improve the thermal stability of blends, especially the initial degradation temperature^37–42^.

**Table 4:** Thermal characteristics of the blends.

Name	T_5%_ (°C)	T_10%_ (°C)	T_20%_ (°C)	T_50%_ (°C)	T_Max1_ (°C)	T_Max2_ (°C)
B	376 ± 1.1	402 ± 1.3	424 ± 1.5	454 ± 1.1	442 ± 0.7	552 ± 1.7
B/3S-MA	387 ± 0.8	409 ± 0.9	430 ± 1.4	461 ± 0.8	448 ± 1.3	556 ± 1.4
B/6S-MA	390 ± 2.0	410 ± 1.2	431 ± 1.1	462 ± 1.0	448 ± 1.2	555 ± 1.0
B/3E	382 ± 1.2	406 ± 1.0	426 ± 0.9	459 ± 1.5	443 ± 0.8	545 ± 1.6
B/6E	385 ± 1.3	407 ± 0.7	427 ± 1.0	454 ± 1.1	443 ± 0.9	547 ± 1.0

The compatibilizer enriches the interaction and adhesion between phases of a blend. Nonetheless, the bulk of the main phases of a blend does not change. Therefore, the compatibilizer does not have a significant effect on improving the inherent thermal stability of each of the main components of a blend. Despite that, the presence of the compatibilizer in a blend by fining the morphology of the phases makes the longer path for the products of thermal degradation of the weaker phase. As a result, the penetration of the degradation products into the bulk of each of main phases becomes more difficult. Based on the mentioned mechanism, the thermal stability of blends is improved^43^. But to investigate the thermal behavior of the present blends in this research, it seems necessary to consider the role of other factors as well. For instance, the degree of crosslinking of each constituent of the blends and the effect of adding SEBS-MA and EVA on the degree need to be considered. The formation of a chemical crosslink network and its densification increases the thermal stability of cured polymers and delays their thermal degradation^44,45^. Investigating the results of the MDR test and the mechanical properties of the blends proved that adding SEBS-MA to the blends improves the CLD of the blends. Because the presence of 3 phr and 6 phr of SEBS-MA copolymer has increased the CLD of B/3S-MA and B/6S-MA by 5.78 and 8.64 (10^−4^ mol/cm^3^), respectively, compared to B blend. This is while the presence of 3 phr and 6 phr from EVA copolymer has reduced the CLD of B/3E and B/6E by 0.38 and 0.99 (10^−4+^mol/cm^3^), respectively. Also, from the investigation of mechanical properties, it was revealed that the compatibilizing effect of EVA is better; whereas, SEBS-MA mainly improves the properties by increasing the CLD of the blends.

According to the above-mentioned, it can be stated that SEBS-MA improves the thermal stability of the blends more than that of EVA, because it both improves the compatibility between phases and increases the CLD of SEBS. However, EVA plays the role of a compatibilizer better and its presence not only does not improve the CLD of the blends but also reduces it. Therefore, the improvement of thermal properties observed for B/3E and B/6E blends compared to B blend can only be attributed to the improvement of compatibility of the phases in the compatibilized blends via EVA.

### Morphology of the blends

Weak interactions between phases cause the morphology of blends to become coarse. A coarser morphology originates from a lower interface between phases followed by an adverse effect on the properties and performance of a blend. However, the more the interface of phases, the better the transfer of stress from one phase to another occurs, which results to improve properties of the blend. The presence of the compatibilizer reduces the interfacial tension and increases the interaction and adhesion between the phases, causing finer morphology, improving the interface, and generally increasing the properties of the blends^46–48^.

**Figure 7:**
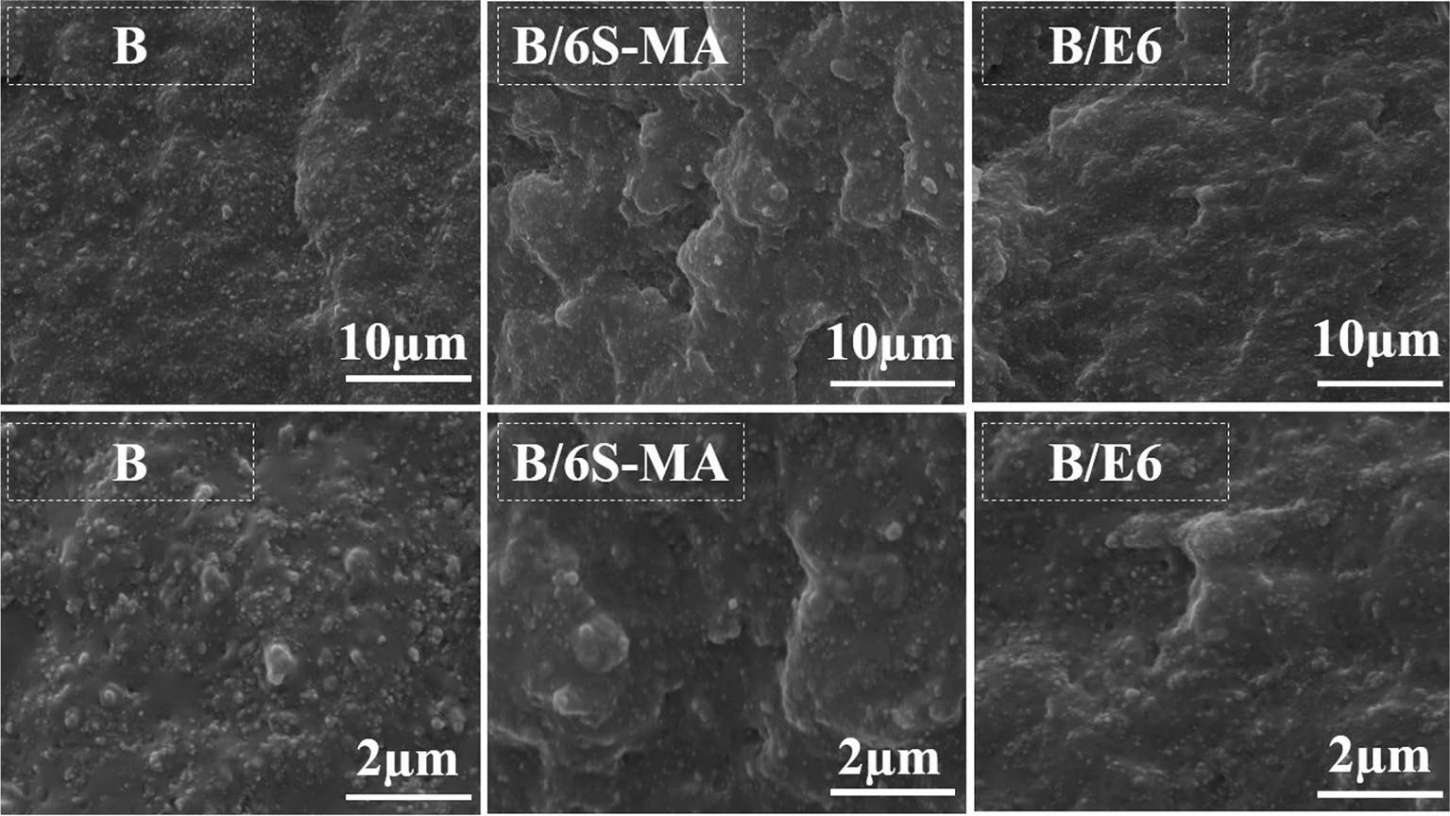
SEM micrographs of the cryo-fractured surface of the blends.

Figure 7 compares the cryo-fractured surfaces of B/6S-MA and B/6E Compatibilized blends with B blend in two magnifications. It can be seen from Fig. 7 that B blend has a coarse and non-uniform morphology in the absence of the compatibilizer. Lumpy structures with irregular shape surfaces indicate no strong interaction between phases. The comparison of morphological images in Fig. 7 demonstrates that B/6S-MA and B/6E have a smoother surface and finer morphology than in B sample, and such fine structure is more noticeable in B/E6 blend.

**Figure 8:**
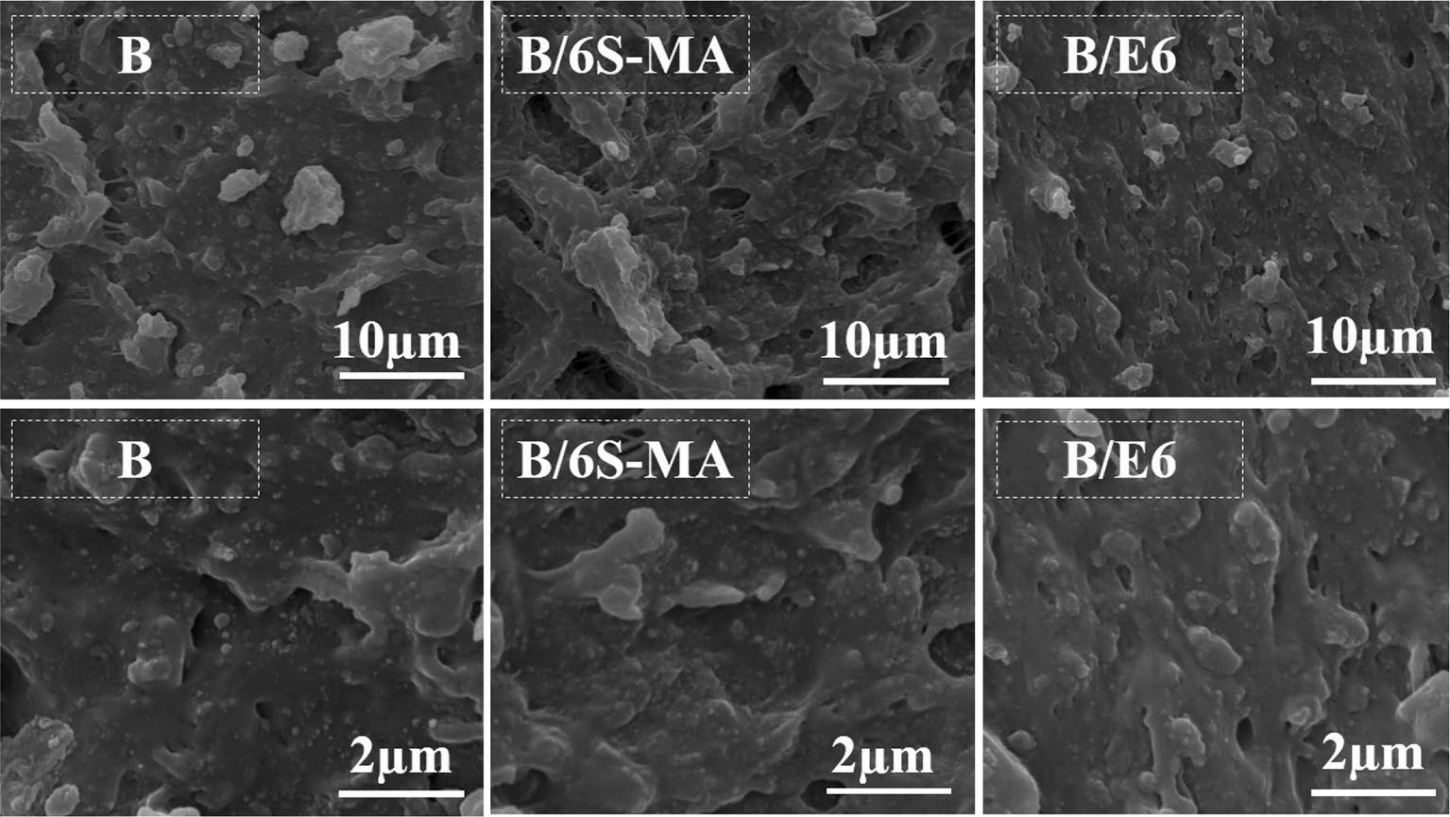
SEM micrographs of the tensile fractured surface of blends.

Figure 8 exhibits the tensile fractured surfaces of B/6S-MA and B/6E blends with B blend. The lumps and holes in B/6E are significantly smaller than B. This indicates that adhesion between phases B/E6 is better than B. In addition, greater adhesion between the phases has made B/E6 more resistant to failure and rupture, which leads the tensile fractured surface of B/6E becomes rougher than the B surface. Likewise, it is seen that the B/6S-MA has a rougher surface than B. Wu et al.^49^ showed that the increase in the CLD of butyl rubber to a certain amount leads to a rougher fractured surface of this polymer. Since B/6S-MA has more CLD than B, its roughness can be attributed to the higher CLD. The results obtained from Figs. 7 and 8 suggest that EVA could be a better compatibilizer than SEBS-MA. However, the main reason for improving the mechanical and thermal properties of blends in the presence of SEBS-MA is the increasing the blends CLDs by this copolymer.

**Figure 9:**
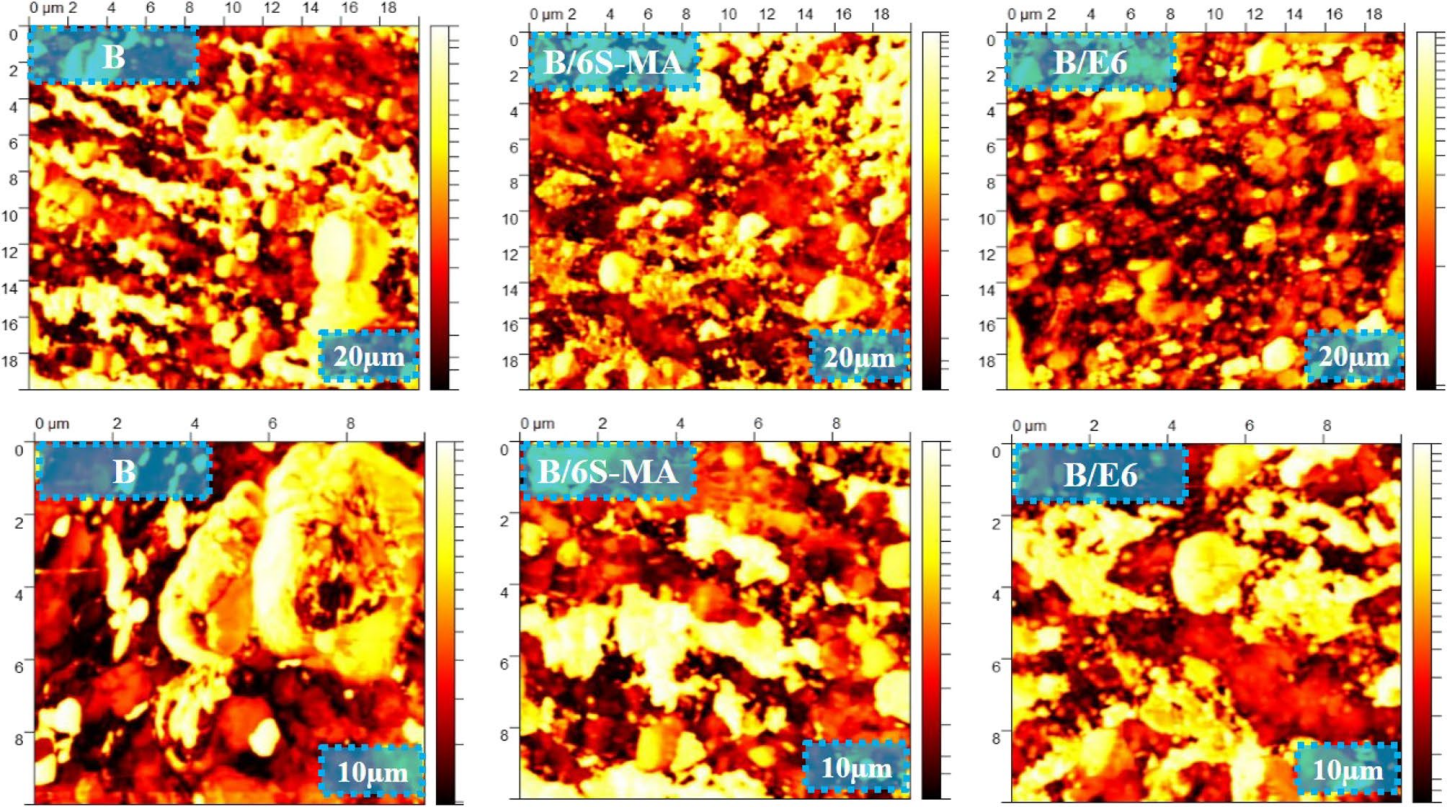
Tapping mode AFM images of the blends.

In order to investigate the morphology of the blends more precisely, AFM is applied. The images in Fig. 9 are obtained by means of AFM. In these images, the harder phase has a lighter color, while the softer phase has a darker color. The cure characteristics results showed that the ΔM of SR were significantly higher than SEBS after curing. Since the CLD of polymers is usually directly related to their hardness, it can be said that SR forms the lighter phase and SEBS forms the darker phase of the blends. The two-phase morphology of the blends can be seen in all three images of Fig. 9. It seems that the morphology of all three blends is Co-continues. It can also be seen that the presence of compatibilizers has made the morphology of the blends finer. However, the morphology of the blend compatibilized with EVA appears to be finer than the other blends. These observations can confirm the results of the SEM test.

### Rubber process analyzer

**Figure 10:**
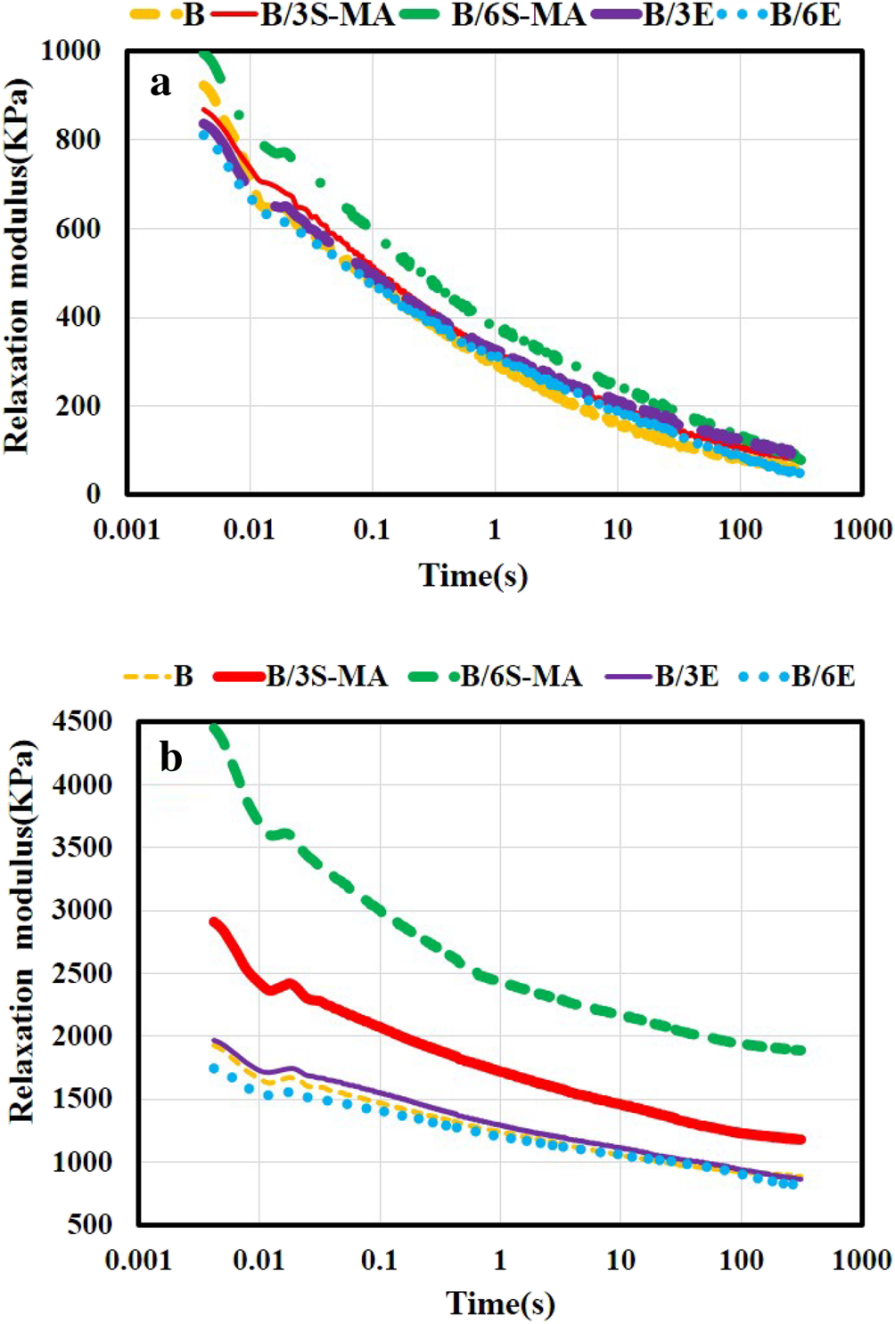
The relaxation modulus versus time for (**a**) uncured and (**b**) cured blends.

The curves of changes in relaxation modulus of the blends, before and after curing, are shown in Fig. 10a, and b, respectively. Based on the data obtained from these curves, the following results are inferred:

- Contrary to the cured blends, it can be seen that the uncured blends completely release the stress.
- The comparison of the initial modulus of the blends before and after curing shows that the modulus of the SEBS-MA compatibilized blends has elevated more than other blends after curing.
- The presence of SEBS-MA and increasing its amount result in the greater relaxation modulus of blends.
- The presence of 6 phr EVA has decreased the relaxation modulus of the blend. In comparison the presence of 3 phr EVA results in a minor reduction in the relaxation modulus of the blend, while.

As illustrated in Fig. 10, contrary to the cured blends, the uncured blends have completely released from the applied stress and have a final relaxation modulus value close to zero. This behavior is due to the fact that in the cured blends, the formation of a crosslinked network dramatically reduces the mobility of the chains and prevents the complete relaxation of the polymer chains. But before curing, due to the lack of crosslinks, the movement of the chains faces many fewer restrictions^50,51^. Also, curing the blends has caused a prominent increase in their relaxation modulus. It is worth noting that the relaxation modulus of the SEBS-MA compatibilized blends is much higher than that of the EVA compatibilized blends and the un-compatibilized blend (B). For example, the initial modulus of B/6S-MA, B/6E, and B blends in the uncured state is 1058, 846, and 966 kPa, respectively, and after curing, it reaches 4872, 1845, and 2061 kPa, respectively. Based on the results obtained from the MDR test, most of the improvement of modulus in the SEBS-MA compatibilized blends is related to the positive effect of SEBS-MA on the curing characteristics of the blends. The increment in modulus after curing is due mainly to the positive effect of SEBS-MA on increasing the CLD of the blends. Moreover, in the uncured state, SEBS-MA has increased the modulus of the blends, which is related to the compatibilizing action of the copolymer.

It has been seen that the presence of a compatibilizer can increase the modulus of the blend^39^. Possibly, the compatibilizer increments the blend modulus by improving the interaction between the blend phases, reducing the dimensions of the phases, and increasing the interface between the phases. Therefore, SEBS-MA through compatibilizing the phases of the blends, enhances the modulus.

Whereas for EVA compatibilized blends it was observed that B/3E modulus is slightly higher than B, but B/6E modulus is slightly lower than B. For an explanation of the trend, the following discussion can be helpful. The stress relaxation experiment has been done at 50 °C. It should be noted that EVA is so softer than SEBS-MA at the temperature of 50 °C, and it causes to diminish the modulus of blends. Therefore, when using the low amount of EVA (3 phr) due to its compatibilization effect, the modulus of blends can increase. When the amount of EVA becomes higher (6 phr), its softening effect becomes prominent, causing a reduction of the modulus of blends. Furthermore, after curing, the impact of EVA on the CLD of the blends is also effective on the modulus. In B/3E blend, due to the lower amount of EVA compared to B/6E blend, the positive effect of compatibility on the modulus is greater than the negative effect of its softening. Nevertheless, in B/6E blend, due to the more amounts of EVA compared to B/3E, the negative effect of EVA softening predominates the positive effect of its compatibility on the modulus of the blends.

### Tear resistance

The results of tear energy of the samples are presented in the Table 5. Un-compatibilized based blend B showed the lowest tear energy among the other blends. Adding compatibilizers to the blend B improves the tear energy. The incorporation of SEBS-MA copolymer improves the tear energy of the blend. As discussed in the previous sections, the crosslink density in the blend with the presence of this copolymer increases (from the results of the curing characteristics and also Table 3) and the possibility of interaction between the two blend components increases (the SEM and AFM images indicated that the morphology of the compatibilized blends has been modified compared to sample B), which result in a higher resistance and improved tear energy. The blends containing EVA copolymer show improved resistance to tear, to the point where using 6% of it as a compatibilizer, the tear energy increases to 17.1 kJ/m^2^, which is the highest value among the other blends. The key role of this copolymer is to improve the interfacial adhesion between the two blend components and to make the finer morphology. Due to the reasons mentioned and because of the inherent properties of EVA, its presence in the blends causes more energy for tearing. Furthermore, when the values are compared, it is revealed that the compatibilizer EVA plays a more effective role in improving the tear energy of the blends compared to SEBS-MA compatibilizer. In addition, tear resistance of the blends increases as content of EVA and SEBS-MA compatibilizers increases.

**Table 5:** Tear energy of the blends.

Name	Tear energy (kJ/m^2^)
B	9.0 ± 0.4
B/3S-MA	11.2 ± 0.1
B/6S-MA	12.3 ± 0.4
B/3E	14.7 ± 0.3
B/6E	17.1 ± 0.8

### Compression set

**Figure 11:**
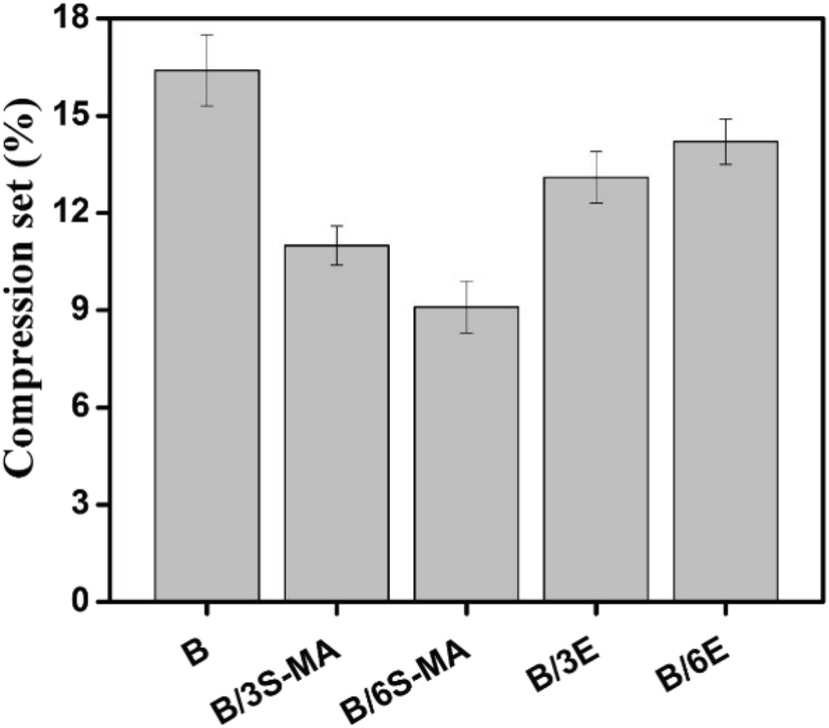
Compression set of the blends.

The Fig. 11 illustrates the compression set of the rubber compounds. Compression set refers to the capability of an elastomer to retain its elasticity and recover its original shape even after being compressed long-time under specific circumstances, revealing a permanent alteration in the elastomer. At elevated temperature, physical and chemical changes in elastomers occur during compression set test and some crosslinks may break. A low compression set indicates good elastic properties and resistance to permanent deformation.

The blends containing EVA copolymer demonstrated lower compression set compared to the based blend B. This could be due to role of EVA as a compatibilizer, creating a finer morphology. Therefore, the interactions between the chains of the two components of the blends in the compatible state are higher, which affects the physic-mechanical behavior of the blends and causes the integrity of the polymers upon compression maintains. To achieve lower compression set, the optimal amount of EVA is 3%, and further loading of EVA does not result in lower compression set, due to its inherent durability upon compression.

The addition of the SEBS-MA compatibilizer to the blends leads to a noticeable decrease in compression set, with reductions of up to 10%. According to discussion in earlier sections, the presence of the copolymer during the curing reaction enhances the crosslinking and affects the microstructure, improving the interaction of the blend components and finer morphology, resulting in improved polymer recovery. Consequently, the decrease in compression set is attributed to this phenomenon, which in other words, causes a significant increase in the ability of the SEBS-MA compatibilized blends to achieve a maximum strain recovery and durability after the compression set experiment.

## Conclusion

In this work, for the first time, the SR/SEBS blends are compatibilized with SEBS-MA and EVA copolymers, and then cure characteristics, morphology, relaxation modulus, as well as tensile and thermal properties of blends are investigated. The results of various tests and analyses shows that both EVA and SEBS-MA copolymers can enhance the properties and performance of SR/SEBS blends by improving the compatibility. In the SEBS-MA compatibilized blends, cure rate and ΔM increase with rising SEBS-MA content, but t_s2_ and optimum cure time decrease. For Instance, the presence of 6 phr SEBS-MA causes the value of ΔM elevates from 14.40 dN m to reach 20.73 dN m. However, the effect of EVA copolymer on the curing behavior of blends does not follow a specified trend. The comparison of the mechanical properties of the blends demonstrates that the EVA compatibilized blends have higher tensile strength and strain at break than the SEBS-MA compatibilized blends. However, the modulus of SEBS-MA compatibilized blends is higher than other blends. The higher modulus of SEBS-MA compatibilized blends is attributed to their higher CLD. Investigation of the thermal properties of the blends confirms that the compatibilized blends have higher thermal stability than the un-compatibilized blend (B). Among the investigated samples, the SEBS-MA compatibilized blends have the best thermal properties. This observation is also attributed to the higher CLD of SEBS-MA compatibilized blends. SEM and AFM images show the morphological improvement of the compatibilized blends. However, the EVA-compatibilized blend (B/6E) has finer morphology, which indicates that EVA has been more successful than SEBS-MA in improving phase compatibility. The investigation of the data obtained from the RPA confirms the results of other tests. Based on the comparison of the properties and characteristics of the prepared samples, it can be concluded that SEBS-MA, while compatibilizes the blends, enhances the properties of the blends mainly by rising the CLD of the SEBS phase. Whereas EVA promotes the characteristics and properties of the blends mainly by increasing the compatibility between the different phases of the blend. Therefore, each of these two copolymers enhances the properties of the host blends through their responsive mechanism. Furthermore, the compatibilized blends revealed promoted tear resistance and better durability after compression compared to the un-compatibilized blend.

## Data Availability

The datasets used and/or analyzed during the current study are available from the corresponding author on reasonable request.
